# *Henricia djakonovi* sp. nov. (Echinodermata, Echinasteridae): a new sea star species from the Sea of Japan

**DOI:** 10.7717/peerj.2863

**Published:** 2017-01-10

**Authors:** Anton Chichvarkhin

**Affiliations:** National Scientific Center of Marine Biology, Far Eastern Branch of Russian Academy of Sciences, Vladivostok, Russia; Far Eastern Federal University, Vladivostok, Russia

**Keywords:** Sea of Japan, East sea, New species, Asteroidea, Spinulosida

## Abstract

A new sea star species, *H. djakonovi* sp.n., was discovered in Rudnaya Bay in the Sea of Japan. This is a sympatric species of the well-known and common species *Henricia pseudoleviuscula* Djakonov, 1958. Both species are similar in body size and proportions, shape of skeletal plates, and life coloration, which distinguishes them from the other *Henricia* species inhabiting the Sea of Japan. Nevertheless, these species can be distinguished by their abactinal spines: in both species, they are short and barrel-like, but the new species is the only *Henricia* species in Russian waters of the Pacific that possesses such spines with a massive, smooth, bullet-like tip. The spines in *H. pseudoleviuscula* are crowned with a variable number of well-developed thorns. About half (<50%) of the abactinal pseudopaxillae in the new species are oval, not crescent-shaped as in *H. pseudoleviuscula*.

## Introduction

The sea stars of the genus *Henricia* Gray, 1840 (blood stars) belonging to the family Echinasteridae (Asteroidea, Spinulosida) are a group of organisms with poorly developed systematics despite their wide distribution and abundance in the world’s oceans, especially in the northern Pacific (Verrill, 1909; Verrill, 1914; Fisher, 1911; Fisher, 1928; Fisher, 1930; Djakonov, 1961; Clark et al., 2015). The first systematic accounts of this genus of the North Pacific were undertaken early in the 20th century (Clark, 1901; Verrill, 1909; Verrill, 1914; Fisher, 1911; Fisher, 1928; Fisher, 1930) by American scholars. Later Japanese and Russian authors further contributed to the knowledge of Asian fauna with the description of several new taxa (Hayashi, 1940; Djakonov, 1949; Djakonov, 1950; Djakonov, 1958; Djakonov, 1961). However, in his last review of *Henricia*, Djakonov (1961) emphasized that *“current systematics of the genus are a very complicated task, imperfect, and required further detailed investigation.”*

Studies on North Pacific *Henricia* were few until 2010 when *Henricia* from the Aleutian Islands were reassessed (Clark & Jewett, 2010; Clark & Jewett, 2015), and 13 new species were described. In the same year, Eernisse, Strathmann & Strathmann (2010) with co-authors described a new species, a brooder inhabiting the North American shore from Alaska to Baja California, and they restricted the geographic distribution of *Henricia leviuscula* (Stimpson, 1857), a name that has been widely applied to *Henricia* in the North Pacific, to only a portion of the cool temperate coastline of the western North America.

More recent reports on *Henricia* in the Asian fauna were also published (Hayashi, 1973; Rho & Shin, 1980; Shigei, 1991; Shin & Rho, 1996; Xiao, Liao & Liu, 2011; Shin & Ubagan, 2015), but in some cases, it remains unclear what characters were used for identification of those specimens. Meanwhile, some Russian scholars (Yavnov, 2010; Dautov & Tyurin, 2014; Lebedev, 2015) summarized literature data. In two of these works (Dautov & Tyurin, 2014; Lebedev, 2015), the echinasterids are represented by only “*Henricia* sp.,” while the atlas by Yavnov (2010) contains multiple questionable identifications, with most images lacking data that would be necessary for appropriate species identification.

To date, the fauna of the echinasterids of the Russian Pacific seas totals 28 nominal species belonging to *Henricia* and *Aleutihenricia* (Smirnov, 2013). This number is based merely on Djakonov’s publications and taxonomical rearrangements by Clark & Jewett (2010), therefore the actual species abundance in this region is still in need of study. Recent studies have demonstrated that using field observations including life coloration and wide range of morphological data analyses allow the discovery of previously overlooked sea star species (e.g., Clark & Jewett, 2010; Clark & Jewett, 2011; Clark & Jewett, 2015; Eernisse, Strathmann & Strathmann, 2010).

This study reports the discovery of a new *Henricia* species that is well distinguished by morphology from the other species of this genus found in the Sea of Japan. The only similar species in this region is *H. pseudoleviuscula* Djakonov, 1958, which possesses a similar size, and occupies the same habitats. The distribution of *H. pseudoleviuscula* is restricted to the northwestern part of the Sea of Japan (Djakonov, 1961; Yavnov, 2010), where it is one of the most abundant echinasterids (my personal observation: of 272 *Henricia* specimens collected in Vostok Bay in July and August 2016, 218 were identified as *H. pseudoleviuscula*). A similar species has not been reported from outside the Russian waters in Japan and Korea (Hayashi, 1940; Hayashi, 1973; Rho & Shin, 1980; Shigei, 1991; Shin & Rho, 1996; Xiao, Liao & Liu, 2011; Shin & Ubagan, 2015). In earlier literature, *H. djakonovi* may have been referred to as *H. leviuscula*, with which it shared a similar appearance. Its restricted distribution range may explain the unavailability of this species in early collections.

Discovery of a new and previously overlooked species in Far Eastern seas of Russia is to be expected in rocky nearshore areas because most previous collections by Russian expeditions in this region were done by trawling rather far from shore on soft bottoms. In general, near-coastal rocky bottoms accessible by SCUBA-diving (the main habitats for *Henricia* diversity) remain poorly studied in the Northwestern Pacific, as recent studies have demonstrated (Chichvarkhin, Chichvarkhina & Kartavtsev, 2016; Chichvarkhin et al., 2016).

## Materials and Methods

Sea stars were collected by SCUBA-diving in Rudnaya, Kievka, and Vostok bays in the Sea of Japan during 2015–2016, and the animals were preserved in 96% ethanol. The specimens were deposited in the collection of the Museum of National Scientific Center of Marine Biology, Vladivostok, Russia (MIMB). Life coloration, abactinal skeletal reticulation, and spines shape were checked in 25 and 218 specimens of *H. pseudoleviuscula* from Rudnaya Bay (October, 2015) and Vostok Bay (July & August, 2016), respectively. These animals were released within several hours into the natural environment at the same sites where they were collected. Sea star collection is not regulated by Russian law. They were not collected in protected waters. The images were taken using a Nikon D7000 camera and a Nikon Nikkor 60 f2.8 lens. Skeletal spines were cleaned from soft tissues, and skeletal plates were denuded with sodium hypochlorite. Scanning electron images of the spines were obtained by using a Zeiss Sigma electron microscope after carbon coating.

The electronic version of this article in Portable Document Format (PDF) will represent a published work according to the International Commission on Zoological Nomenclature (ICZN), and hence the new names contained in the electronic version are effectively published under that code from the electronic edition alone. This published work and the nomenclatural acts it contains have been registered in ZooBank, the online registration system for the ICZN. The ZooBank LSIDs (Life Science Identifiers) can be resolved and the associated information viewed through any standard web browser by appending the LSID to the prefix http://zoobank.org/. The LSID for this publication is: urn:lsid:zoobank.org:pub:632d0662-da5f-4f7e-a63a-f112de99a7d5. The online version of this work is archived and available from the following digital repositories: PeerJ, PubMed Central and CLOCKSS.

## Results and Discussion

**Table:** 

**Figure 1:**
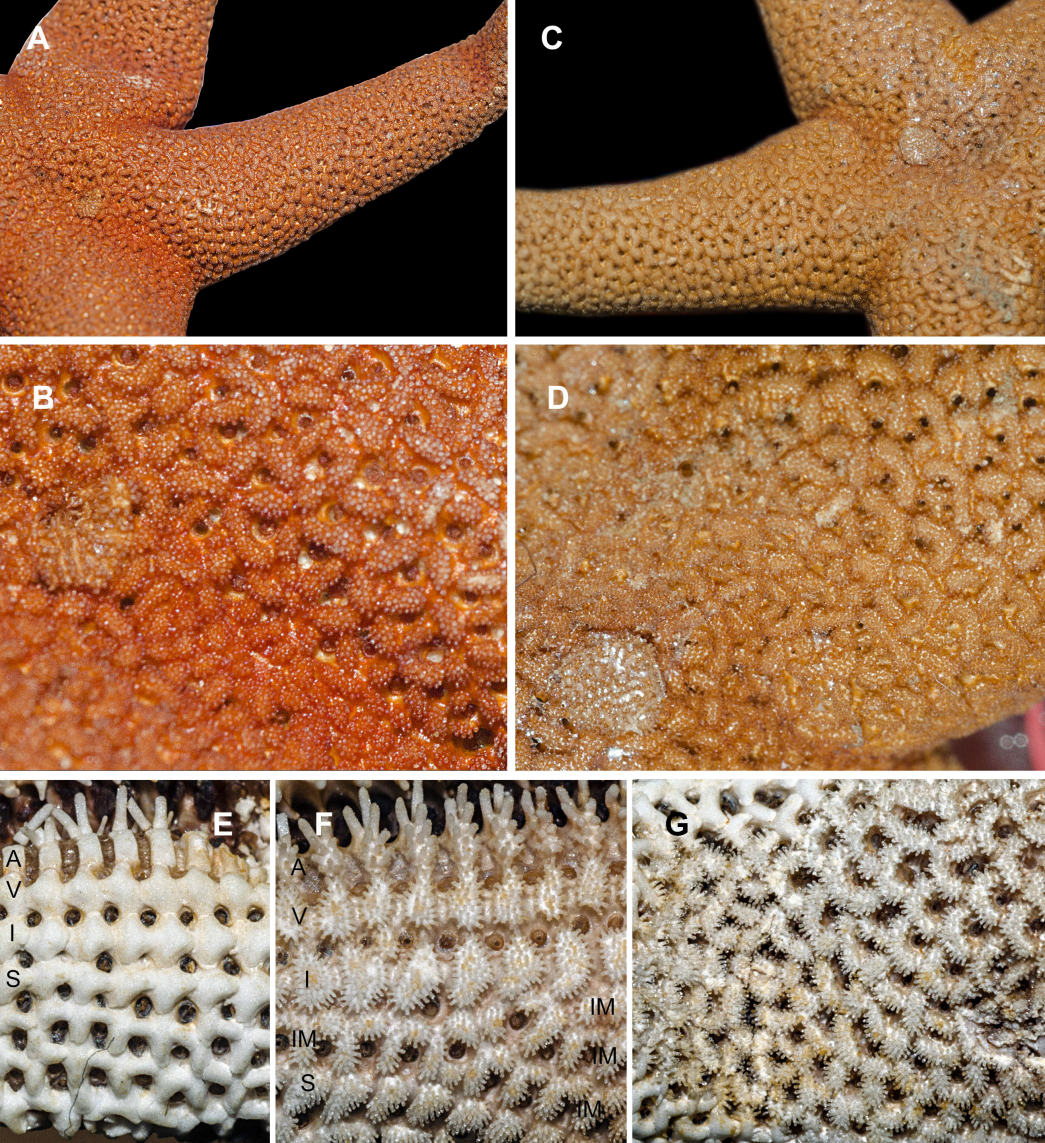
Abactinal side of *Henricia* spp., preserved specimens. (A, B) *H. djakonovi*, holotype. (C, D) *H. pseudoleviuscula*, Rudnaya Bay. (E) *H. djakonovi*, actinal side plates. (F) *H. djakonovi*, actinal side pseudopaxillae. (G) *H. djakonovi*, abactinal side pseudopaxillae. Abbreviations: A, adambulacral plates; V, ventrolateral plates; I, inferomarginal plates; Im, Intermarginal platess; S, superomarginal plates.

**Figure 2:**
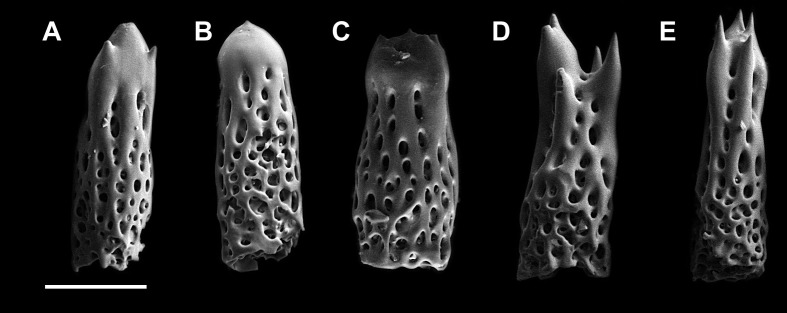
Abactibal spines of the paxillae located at the base of ray. (A–C) *H. djakonovi*, holotype. (D, E) *H. pseudoleviuscula*, Rudnaya Bay. Scale 100 µm.

**Figure 3:**
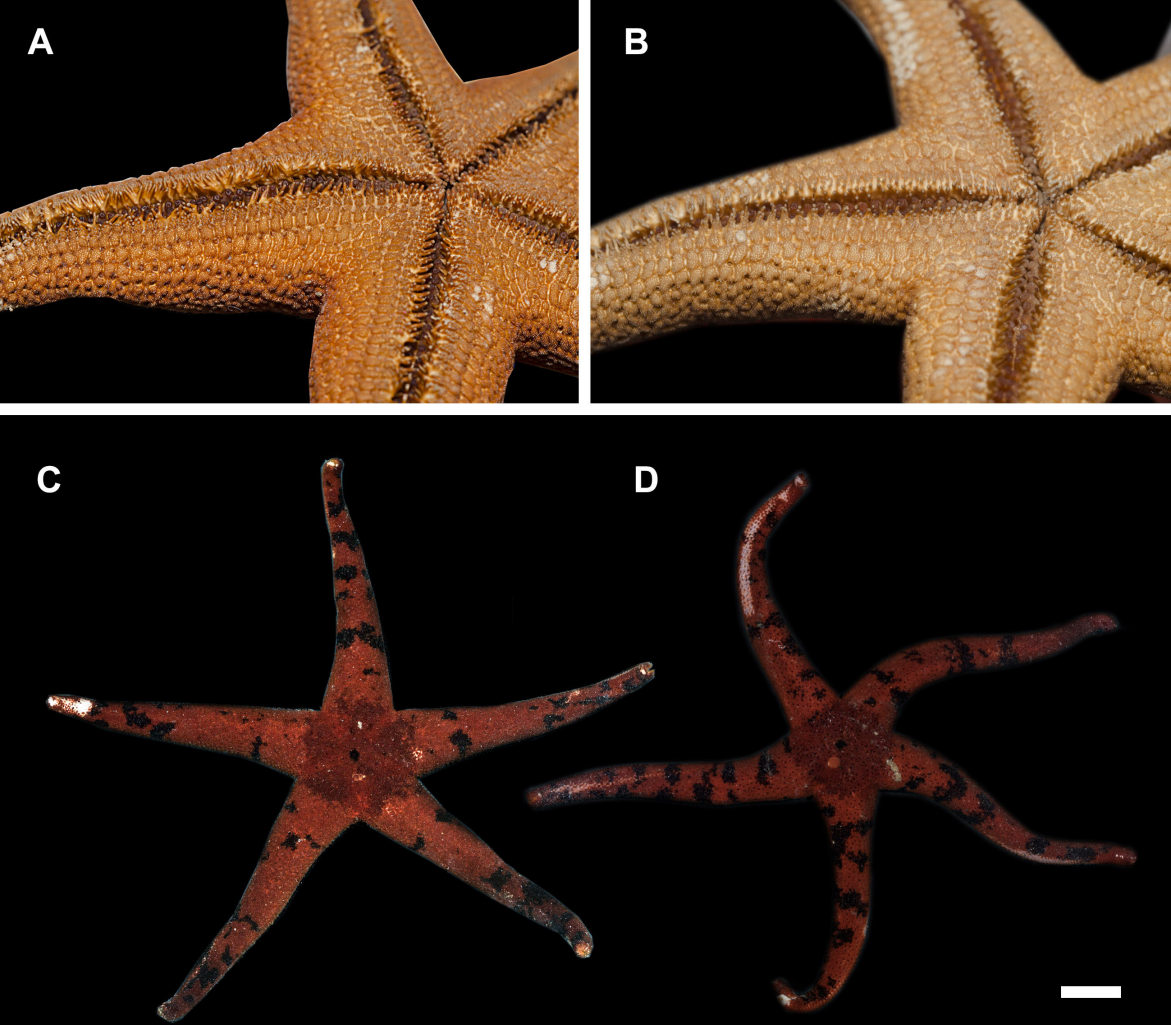
Actinal side of preserved and live specimens. (A) *H. djakonovi*, holotype, (B) *H. pseudoleviuscula*, *Rudnaya Bay.* (C) *H. djakonovi*, holotype, (D) *H. pseudoleviuscula*, Rudnaya Bay. Scale bar: 10 mm.

**Figure 4:**
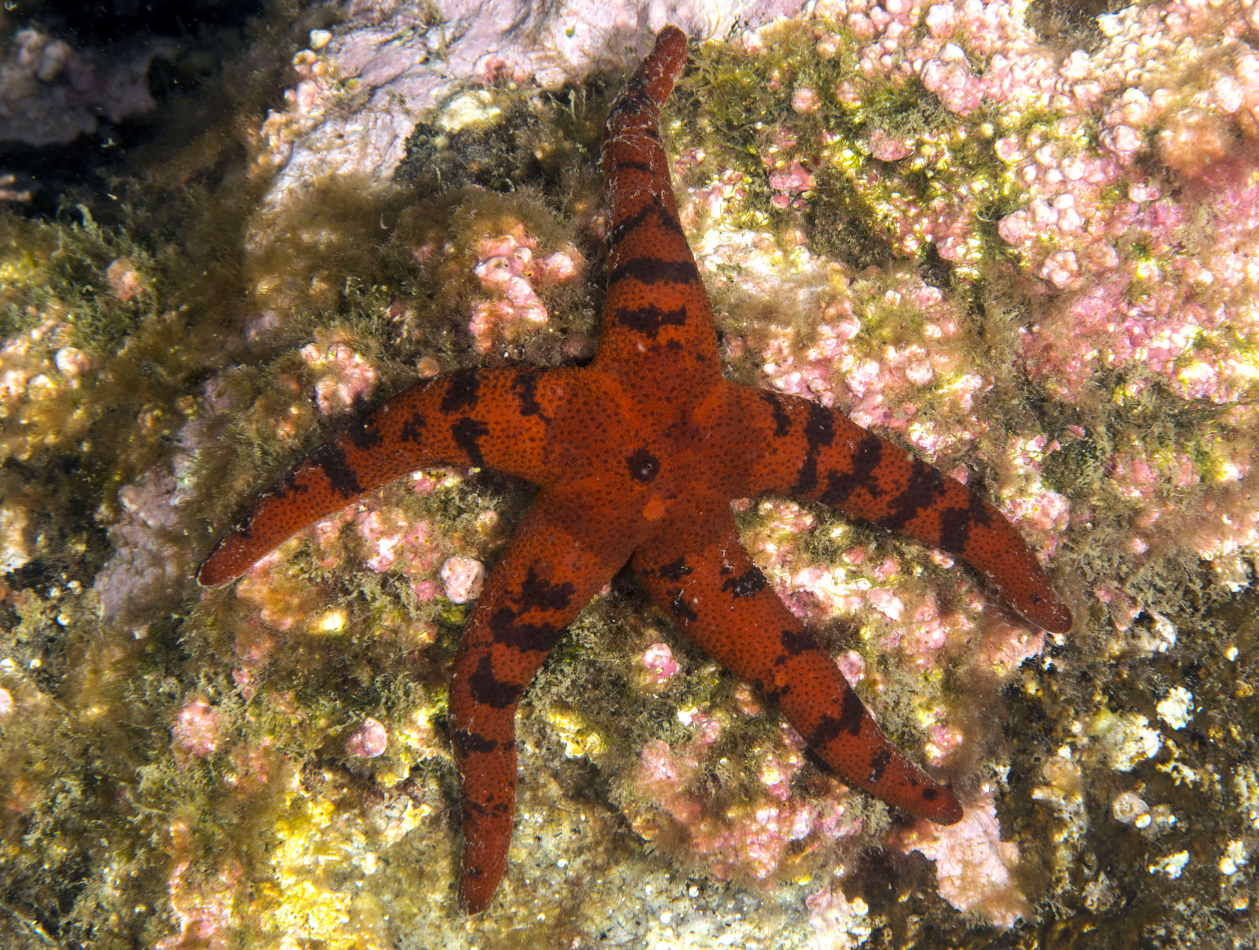
*H. djakonovi* in natural environment, Rudnaya Bay, 5 Oct 2015 (specimen not preserved).

Type Echinodermata Bruguière, 1791 [ex Klein, 1734]
Class Asteroidea de Blainville, 1830
Order Spinulosida Perrier, 1884
Family Echinasteridae Verrill, 1870
Genus *Henricia* Gray, 1840, type species *Asterias oculata* Pennant, 1777.
*Henricia djakonovi* sp. nov. (Figs. 1A, 1B; 2A, 2C; 3A–3C; 4)
ZooBank LSID: urn:lsid:zoobank.org:act:2B39F3DC-83EE-45B3-976B-AC1B7B058957

### Type material

Holotype. *MIMB331294* 4 Jun 2016, Senkina Shapka pinnacle, Rudnaya Bay, 44.36°N 135.83°E, 16 m, leg. A. Chichvarkhin. Paratype. *MIMB331304* 4 Jun 2016, Senkina Shapka pinnacle, Rudnaya Bay, 44.36°N 135.83°E, 16 m, leg. A. Chichvarkhin.

### Type locality

Senkina Shapka pinnacle, Rudnaya Bay, NW Sea of Japan.

### Examined material

*Henricia pseudoleviuscula*: *MIMB331271* specimen4 Jun 2016, Senkina Shapka pinnacle, Rudnaya Bay, 44.36°N 135.83°E, 16 m, leg. A. Chichvarkhin; 2 specimens 5 Oct 2015, Senkina Shapka pinnacle, Rudnaya Bay,44.36°N 135.83°E, 15–18 m, leg. A. Chichvarkhin; *MIMB33126*1 specimen 27 Jun 2015, Kievka Bay, Skaly Is., 5 m, leg. A. Chichvarkhin; *MIMB33128, MIMB33131*11specimens 23–25 Aug 2015, Vostok Bay, leg.K. Dudka; *ZIHU-2397* (Hokkaido University) 2 specimens *H. reniossa* syntypes (marked as a ‘paratype’) 30 Sep 1906, Albatross station 5031 Bomase’ri Shima.

### Diagnosis

R to 7 cm; disc relatively large, rays long, fairly rigid, marginal and ventrolateral plates are similar, pillow-shaped; about a half of abactinal plates cross-shaped, arranged as roof tiles: each plate’s proximal outgrowths cover adjacent proximal plate; elevated sides of cross-shaped plates form crescent-shaped pseudopaxillae with 20–30 spines, the other triangular or irregular-shaped abactinal plates lacking proximal outgrowths form oval pseudopaxillae; abactinal plates on disk close-set but not very tightly leaving space for papular areas; more than one intermarginal row, the longest intermarginal row contains 20 plates; color in life dark/dirty red with almost black spots and wide transversal lines; aboral side of disk dark, brownish-red divided into five triangular sectors with lighter lines connecting anal pore and disk margin. Abactinal and marginal spines blunt with rounded droplet-like apex, some apices bear few very short thorns.

### Description

External morphology. R:r 4.7–5, R of holotype is 52 mm, *r* = 11*mm*; *R* = 38 mm, *r* = 7.5 mm in paratype. The rays slender not very slim, slightly swollen at base, tapering to blunt tips. Most abactinal plates (>50%) cross-shaped with two proximal outgrowths covering adjacent plates, their elevated proximal sides form crescent-shaped pseudopaxillae. The other plates triangular or irregular shaped, lacking proximal outgrowths, form round and oval pseudopaxillae. Plates convex without ridges or tubercles. Abactinal surface (Fig. 1A) thick, rigid; abactinal plates on disk close-set forming tight reticulation, leaving space for papular areas. Abactinal pseudopaxillae (Fig. 3G) bear 20–30 spines, but some smaller pseudopaxillae bear 8–10 spines. Papular areas nearly twice smaller than average abactinal pseudopaxilla, includes one or two papulae. Abactinal spines short, stout, 200–240 µm long, 60–100 µm wide, barrel-shaped, apex smooth, massive (Fig. 2A–C). The apex of most spines massive, round, resembles glass droplet. Some spines bear 1–4 very short thick apical thorns. Smooth shape of the spines results in droplets-covered appearance of pseudopaxillae in living and preserved specimens.

Superomarginal row bent dorsally at ray base (Fig. 3A), consists of square pillow-shaped plates. Inferomarginals and ventrolaterals are similarly pillow-shaped. Five or six intermarginal rows consisting of irregular shaped plates constitute a proximal intermarginal area, which provides inflation of ray base, often poorly discernible (Fig. 3A). Longest intermarginal row is adjacent to superomarginal row, bent dorsally, consists of 20 plates, extends from ray base beyond 1/3 of ray length. The other intermarginal rows irregular, consisting of 3–13 plates. Superomarginals bear 20–35 smooth-ended spines, same as abactinal spines, arranged in 4 rows. Inferomarginal row extended over entire ray length (Fig. 1F), its pseudopaxillae bear 30–45 spines in 4–6 transversal rows.

Ventrolateral pseudopaxillae bear 25–30 thorny spines in 4–5 rows. Adambulacrals with 10–11 spines in two transversal rows bearing numerous thorns on apices; 3–4 larger adambulacral spines located near furrow; size of the other spines facing ventrally gradually decreases outwards furrow (Fig. 3F). Deep furrow spine small, single. Oral plates not fused, each bear single apical, four marginal, and four suboral blunt spines.

Color in life. Rays orange-red, abactinal side of the disk dark, divided in five triangular sectors with lighter stripes connecting the center of disk (anal pore) and disk margin (armpits) (Figs. 3C and 4). Anal area almost black; madreporite partially creamy, partially brownish. In ethanol-preserved specimens, the madreporite is the same color as abactinal side of disk. Abactinal side of rays marked with dark brown to black spots of irregular shape, some of them look like wide transversal lines. Actinal side is uniformly colored orange-red, same as abactinal background color of rays.

### Ecology

The species was found on solid rock at the depths of 14–18 m at water temperature of 2–6°C.

### Etymology

The name is dedicated to AM Djakonov, the famous Russian (Soviet) echinoderm taxonomist who described several species in the genus *Henricia*.

## Discussion

*Henricia djakonovi* sp. n.is superficially very similar to sympatric *H. pseudoleviuscula* Djakonov, 1961, with which it shares the same habitat, and it may be distinguished by (1) brown abactinal side of the disk divided into five triangular sectors, while the abactinal side of the disk of *H. pseudoleviuscula* is solid brown (Figs. 1C, 1D and 2D); (2) several intermarginal rows, the longest one consisting of 20 plates, while single rudimentary intermarginal row of *H. pseudoleviuscula* is shorter, consists of 10-16 plates (Fig. 2C); (3) the plates on abactinal side of disk near the madreporite of *H. pseudoleviuscula* are set very tightly, almost fused lacking papular areas between, while the plates of *H. djakonovi* form a mesh with papular areas (Figs. 1B, 1D and 4) ; (4). Almost all abactinal plates of *H. pseudoleviuscula* are cross-shaped forming crescent-shaped pseudopaxillae, while only a half of the abactinal plates of *H. djakonovi* are cross-shaped, the other plates are oval and round; (5) the abactinal spines of *H. djakonovi* are with massive, smooth droplet-shaped tips, while *H. pseudoleviuscula* has spines with the apices bearing 3–10 well discernible thorns (Figs. 3D and 3E).

*Henricia djakonovi* sp. n. and *H. pseudoleviuscula* can be easily distinguished from the other *Henricia* species by their spotted live coloration. The ventrolateral and marginal plates of *H. reniossa* Hayashi, 1940 represented by Hayashi (1940, Fig. 9) are similar to *H. pseudoleviuscula* and *H. djakonovi*, although the type specimens of *H. reniossa* differ from Hayashi’s images: their ventrolaterals, infero- and superomarginals are cross-shaped. Also, the abactinal spines of *H. reniossa* type specimens are different being club-shaped and bearing numerous long thorns. Few additional intermarginal rows are reported for *H. reniossa asiatica* Djakonov, 1958 (Djakonov, 1958; Djakonov, 1961), although it can be distinguished by more numerous spines on adambulacral plates, a short additional ventrolateral row, and longer rays (R:r = 6–10). The inferomarginal plates in both *H. djakonovi* and *H. pseudoleviuscula* are subquadratic, do not possess a ridge, while in *H. reniossa* these plates are transversally elongated possessing a very distinctive ridge.

In total, 243 spotted *Henricia* specimens examined in the field in Vostok and Rudnaya Bays were unequivocally identified as *H. pseudoleviuscula* by their solid abactinal disk coloration, thorny spines, lack of papulae in the center of the disk, and single short intermarginal row. At least 10 individuals of *H. djakonovi* were found in the wild and from underwater images (Fig. 4) on Senkina Shapka in 2012–2015, but most of these specimens were not preserved. No transitional forms comprising a combined set of diagnostic characters of both species were found.

## Additional Information and Declarations

### Field Study Permissions

The following information was supplied relating to field study approvals (i.e., approving body and any reference numbers): Only fishery and endangered objects are regulated. Sea stars are considered aquaculture pests and their collection is not regulated. They were not collected in protected waters.

### Data Availability

The following information was supplied regarding data availability: The raw data is contained in the figures.

### New Species Registration

The following information was supplied regarding the registration of a newly described species: Henricia djakonovi Taxon LSID: urn:lsid:zoobank.org:act:2B39F3DC-83EE-45B3-976B-AC1B7B058957 Publication LSID: urn:lsid:zoobank.org:pub:632d0662-da5f-4f7e-a63a-f112de99a7d5.
